# Loading Graphene Quantum Dots into Optical-Magneto Nanoparticles for Real-Time Tracking *In Vivo*

**DOI:** 10.3390/ma12132191

**Published:** 2019-07-08

**Authors:** Yu Wang, Nan Xu, Yongkai He, Jingyun Wang, Dan Wang, Qin Gao, Siyu Xie, Yage Li, Ranran Zhang, Qiang Cai

**Affiliations:** 1 State key Laboratory of New Ceramics and Fine Processing, School of Materials Science and Engineering, Tsinghua University, Beijing 100084, China; 2 Key Laboratory of Advanced Materials of Ministry of Education of China, Tsinghua University, Beijing 100084, China; 3 Tsinghua Shenzhen International Graduate School, Tsinghua University, Shenzhen 518055, China; 4 Shenzhen Geim Graphene Center, Tsinghua-Berkeley Shenzhen Institute, Tsinghua University, Shenzhen 518055, China

**Keywords:** GQDs, real-time tracking, optical-magneto nanoparticles, *in vivo*

## Abstract

Fluorescence imaging offers a new approach to visualize real-time details on a cellular level *in vitro* and *in vivo* without radioactive damage. Poor light stability of organic fluorescent dyes makes long-term imaging difficult. Due to their outstanding optical properties and unique structural features, graphene quantum dots (GQDs) are promising in the field of imaging for real-time tracking *in vivo*. At present, GQDs are mainly loaded on the surface of nanoparticles. In this study, we developed an efficient and convenient one-pot method to load GQDs into nanoparticles, leading to longer metabolic processes in blood and increased delivery of GQDs to tumors. Optical-magneto ferroferric oxide@polypyrrole (Fe_3_O_4_@PPy) core-shell nanoparticles were chosen for their potential use in cancer therapy. The *in vivo* results demonstrated that by loading GQDs, it was possible to monitor the distribution and metabolism of nanoparticles. This study provided new insights into the application of GQDs in long-term *in vivo* real-time tracking.

## 1. Introduction

Cancer is known to be one of the leading causes of death in almost every country of the world [1]. The ideal therapy for cancer is to deliver suitable treatment (chemotherapeutic drugs, genetic drugs, nanoparticles, etc.) to the right place at the right time. Various delivery systems are designed to track the release and infiltration process of nanoparticles or drugs at the tumor site, guiding local treatment and monitoring the effects after treatment [2,3,4]. Due to low temporal or spatial resolution, the contemporary methods available are unable to track the kinetics of drugs or nanoparticles *in vivo* for a sustained period of time [5]. Optical imaging presents non-ionizing, non-invasive, and non-destructive features with high precision and efficiency [6]. Small molecule organic dyes, including near-infrared fluorescent dyes sulfo-cyanine7 (Cy7), indocyanine green (ICG), Dye800 and green fluorescent dyes fluorescein isothiocyanate (FITC), are employed as imaging probes for their excellent luminescence, while properties such as poor light stability severely limit their biomedical applications [7,8]. Semiconductor quantum dots have excellent luminescence and light stability, and are considered as alternatives to organic dyes. However, high toxicity and poor water solubility would hinder their application in biomedical fields. Moreover, the relatively large size of semiconductor quantum dots and the scintillating fluorescence are both obstacles to successful imaging at the molecular level [9]. Therefore, the development of new fluorescent materials is of great significance to biomedical imaging. 

Graphene quantum dots (GQDs) have drawn more attention in recent years due to their outstanding properties, including excellent light stability and water solubility. Other noteworthy properties of GQDs include their ultra-small size, highly adjustable photoluminescence, as well as excellent multiphoton excitation, high luminosity, and chemical inertness [10,11]. Moreover, various nanomaterials including GQDs and GQD-based nanomaterials are reported to yield reactive oxygen species (ROS) in cells, leading to the promise of photodynamic therapy for cancer [12,13]. GQDs have shown even more promising applications in fluorescence imaging, imaging-guided surgery and biosensing [6,14]. Studies pay close attention to the biocompatibility of GQDs when they are applied in biomedical fields. *In vitro* studies illustrate that GQDs show excellent biocompatibility when co-incubated with various cells; *in vivo* studies also demonstrate the biocompatibility of GQDs [15]. N-doped graphene quantum dots exhibit low cytotoxicity when concentrations are under 200 μg/mL [16]. These studies indicate the promising biomedical applications of GQDs and GQD-based materials.

To achieve multiple functions, it is common to combine GQDs with other nanomaterials. There are many studies on the combination of GQDs and nanomaterials by dipping or sticking GQDs on the surface of nanoparticles for drug delivery and imaging [17,18,19]. Our study proposed an efficient and convenient one-pot approach to load GQDs into Fe_3_O_4_@PPy nanoparticles, which could avoid complicated binders and be easily extended to other nanoparticles. Fe_3_O_4_@PPy nanoparticles were chosen as model nanoparticles since they were proven to be excellent tumor diagnosis and treatment platforms, performing multiple diagnostic functions (such as magnetic resonance imaging and photoacoustic imaging), and have been utilized in various therapies (magnetic hyperthermia and photothermal therapy) [20]. Combination of the GQDs and magnetic nanoparticles enabled the function of real-time tracking in the optical-magneto nanoparticles. Moreover, when loading GQDs into PPy-coated Fe_3_O_4_ nanoparticles, longer metabolic processing in the blood and increased delivery of GQDs were achieved, laying the foundation for the application of GQDs *in vivo*. 

## 2. Materials and Methods

### 2.1. Preparation and Characterization

Polyvinyl alcohol (Sinopharm Chemical Reagent Co., Ltd, Shanghai, China) (PVA, 7.5 g) was dissolved in 100 mL of deionized water. Next, 0.1 g of ferroferric oxide (Sinopharm Chemical Reagent Co., Ltd, Shanghai, China) was dissolved in 10 mL of deionized water and then mixed with 40 mL of PVA solution, followed by 30 minutes of stirring. GQDs (0.15 g) were dissolved in 5 mL of deionized water, then mixed with the above solution and stirred for 30 minutes for uniform dispersion. The synthesis of used OH–GQDs was carried out according to a previous work of Wang et al. [21]. Subsequently, 0.2 mL of pyrrole liquid was slowly added to the above mixed solution and stirred for 10 min. Then, 0.1 g of ferric chloride hexahydrate (Sinopharm Chemical Reagent Co., Ltd, Shanghai, China) was added and stirred at room temperature for three hours. Eventually, the solution was centrifuged five times, washed, and finally dried at 70 °C. The obtained nanoparticles were referred to as GQD-NPs. The as-prepared GQD-NPs were characterized and analyzed by a high resolution transmission electron microscope (HRTEM; JEM-2010F, JEOL, Tokyo, Japan), X-ray diffraction (XRD, Bruker D8 Advance X-ray diffractometer, Bruker, Karlsruhe, Germany), Raman spectroscopy analysis (HORIBA, Montpellier, France), and X-ray photoelectron spectroscopy (XPS; Escalab 250Xi, Thermo Scientific, Waltham, MA USA). Fluorescent spectra were measured at room temperature with a fluorescence spectrometer (FLSP920, Edinburgh Instruments, Edinburgh, UK). Magnetic properties were detected by a vibrating sample magnetometer (Lake Shore VSM 7307, Lakeshore, Columbus, OH, USA).

### 2.2. Cell Toxicity

Murine fibrosarcoma L929 cells were purchased from Peking Union Medical College and cultured in RPMI 1640 basic medium (Gibco, Carlsbad, CA, USA) supplied with 10% fetal bovine serum (Gibco, Carlsbad, CA, USA) and 1% penicillin–streptomycin (Gibco, Carlsbad, CA, USA). Cells were seeded in 96-well plates at 5 × 10^3^ cells per well for 24 h. Then, GQD-NPs with gradient concentrations (50 μg/mL, 100 μg/mL, 160 μg/mL, 250 μg/mL, 500 μg/mL, and 1000 μg/mL) were added into each well. The doses of GQD-NPs were selected according to previous studies [3,22,23,24]. After incubation for 24 h or 48 h, cell viability was measured using a cell counting kit-8 (CCK-8; Dojindo, Kyushu, Japan) following the manufacturer’s instructions. The cell viability values were all normalized to control groups (untreated cells after incubation for 24 h or 48 h). The experiments were conducted in triplicate and the data were expressed as means ± standard deviation. Student’s t-test was used to determine the level of significance. Differences with *p* < 0.05 and *p* < 0.01 were considered statistically significant and highly significant, respectively.

### 2.3. In Vivo Tracking

Animal experiments were conducted under the guidance of the Animal Testing Center of Tsinghua University in accordance with strict animal ethical standards. The BALB/c female mice (5–6 weeks old and 16–18 g) used in our experiments were purchased from Beijing Weitong Lihua Experimental Animal Technology Co., Ltd. (Beijing, China). To establish the tumor model, 0.2 mL human breast cancer cell line MCF-7 cell suspension (5 × 10^6^ cells) was injected subcutaneously into the hind limb of the nude mice. After the tumor grew to 75–100 mm^3^, the nude mice were used in the animal experiments. Each mouse was injected with 0.15 mL of 1 mg mL^−1^ solution intravenously. Subsequently, the mice were anesthetized at 6 h, 24 h, 48 h and then placed in supine position. For image acquisition, the Cellvizio® dual band imaging system (Mauna Kea Technologies, Paris, France) was used to obtain real-time images of the blood vessels (imaged by tail vein injection of Evans blue under 660 nm excitation), and GQD-NPs (imaged under 488 nm excitation) at the tumor site. 

## 3. Results and Discussion

**Figure 1:**
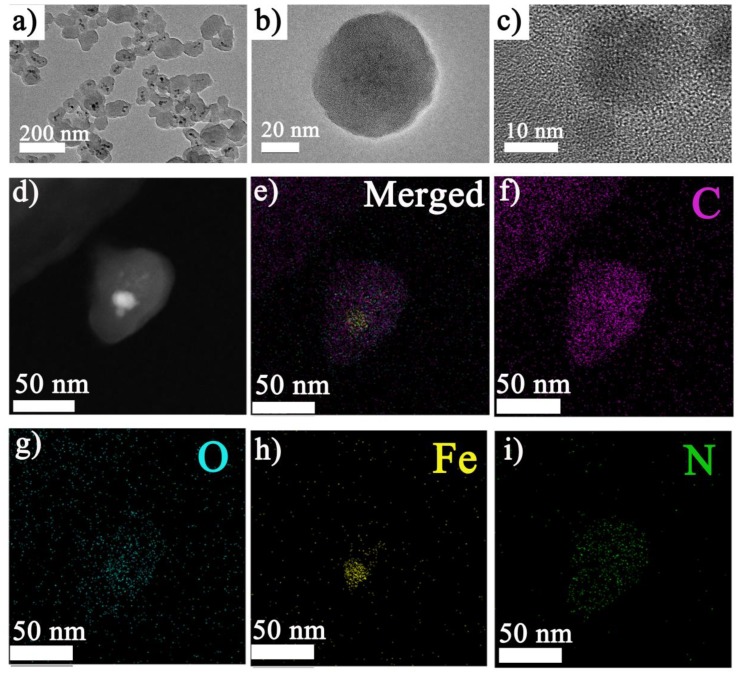
Morphology and elemental analysis of graphene quantum dots (GQDs) loaded nanoparticles: (**a**~**c**) high resolution tranmission electron microscopy (HRTEM) images at different magnifications (bright field image); (**d**~**i**) energy dispersive spectroscopy (EDS) analysis of the nanocomposite; (**d**) graph representation, (**e**) EDS analysis of the nanocomposite (merged), (**f**) EDS C elemental map, (**g**) EDS O elemental map, (**h**) EDS Fe elemental map, (**i**) EDS N elemental map.

The structure of the obtained samples could be clearly seen in the HRTEM image (Figure 1a) since the outside layer and inside nanoparticles had different contrasts. The HRTEM image (shown in Figure 1b,c) further illustrated the amorphous coating of the polymer and the inside nanoparticles with a clear lattice structure. The energy dispersive spectroscopy (EDS) map (Figure 1d–i) demonstrated that the inside nanoparticles mainly consisted of Fe and O, and the outer layer mainly contained C and N. GQDs were hardly identified in the HRTEM and EDS images (Figure S1).

**Figure 2:**
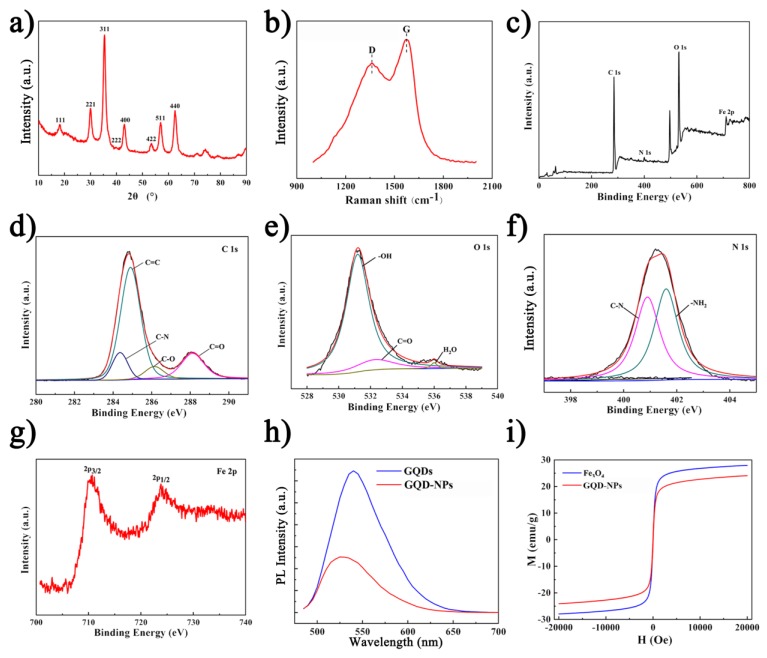
Properties of GQD-nanoparticles (NPs): (**a**) X-ray diffraction (XRD) patterns; (**b**) Raman spectrum, the ordered G band and disordered D band were indicated; (**c**) X-ray photoelectron spectrum (XPS); (**d**–**g**) XPS analysis of C, O, N and Fe, respectively; (**h**) Photoluminescence (PL) spectra with the pulsed laser excitations at 490 nm; (**i**) Magnetizing curve, magnetization (M), magnetic field strength (H).

X-ray diffraction was employed to further validate the content of the nanocomposites. Peaks in Figure 2a were ascribed to Fe_3_O_4_ [3]. The Raman spectrum of the GQD-NPs was shown in Figure 2b. The D peak at 1372 cm^−1^ was generally considered to be the disordered vibration peak of GQDs, caused by lattice vibrations leaving the center of the Brillouin zone, which characterized the defects or edges of the GQDs. The peak present at 1582 cm^−1^ was the G peak, which was a characteristic peak of GQDs. The G peak was higher than the D peak, indicating the more edged structure of the GQDs [21]. The XPS spectra of GQD-NPs were shown in Figure 2c–g. From Figure 2c, it could be seen that the nanocomposites consisted of C, N, O and Fe. The C=C peak of C1s in Figure 2d indicated that there were a large number of conjugated structures in the nanocomposites. The appearance of the C−O peak revealed that there were both amino groups and hydroxyl groups on the surface of GQDs. In the spectrum of O1s in Figure 2e, the C−O and O−H peaks further confirmed the presence of hydroxyl groups on the surface of GQDs. The peak of N–H in Figure 2f was presumed to be the N–H in the nitrogen-containing ring in PPy. The Fe2p in Figure 2g illustrated the presence of Fe_3_O_4_. The XPS spectra further demonstrated that there were Fe_3_O_4_, PPy and GQDs in the nanocomposites [3,21,25]. Studies have shown that excellent fluorescent luminescence performance is one of the most outstanding properties of graphene quantum dots [11,26,27]. Therefore, after loading the GQDs, the optical performance of the nanocomposite would be of concern. As shown in Figure 2h, the GQD-NPs presented an absorption peak under the excitation wavelength of 490 nm, which was the characteristic peak of the GQDs. In Figure 2i, the GQD-NPs retained a slightly decreased saturation magnetization compared to bare Fe_3_O_4_, allowing them to perform as a contrast agent for magnetic resonance imaging. 

**Figure 3:**
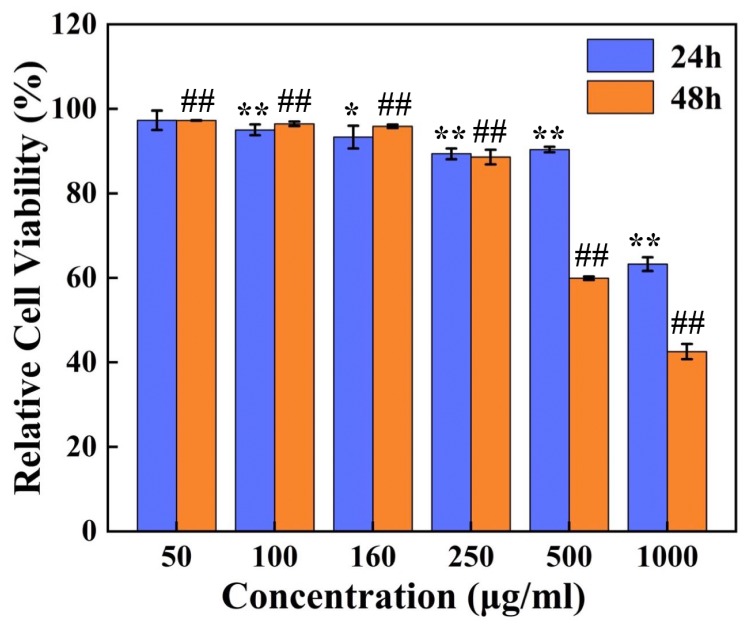
Cell viability of L929 cells incubated with GQD-NPs. The experiments were carried out in triplicate. Data were presented as mean ± standard deviation (SD) (*n* = 3). The cell viability values were all normalized to control groups (untreated cells after incubation for 24 h or 48 h). Asterisk (*) and double asterisks (**) refer to statistical significance of *p* < 0.05 and *p* < 0.01, respectively, compared with control groups between the cell viability values after co-incubation for 24 h; double pounds (##) refer to a statistical significance of *p* < 0.01 compared with control groups between the cell viability values after co-incubation for 48 h.

The viability of L929 cells incubated with gradient concentrations of GQD-NPs (50 μg/mL, 100 μg/mL, 160 μg/mL, 250 μg/mL, 500 μg/mL, and 1000 μg/mL) for 24 h or 48 h was detected by CCK8. As shown in Figure 3, after co-incubation for 24 h, there were significant differences between the groups whose concentrations of GQD-NPs were higher than 50 μg/mL and the control group; after co-incubation for 48 h, high significant differences were found between all the groups treated with GQD-NPs and the control group. The outcome illustrated that GQD-NPs exhibited excellent biocompatibility when the concentration was lower than 250 μg/mL. This particular outcome was in line with previous studies which investigated the cell toxicity of GQDs [28] or Fe_3_O_4_@PPy [22].

**Figure 4:**
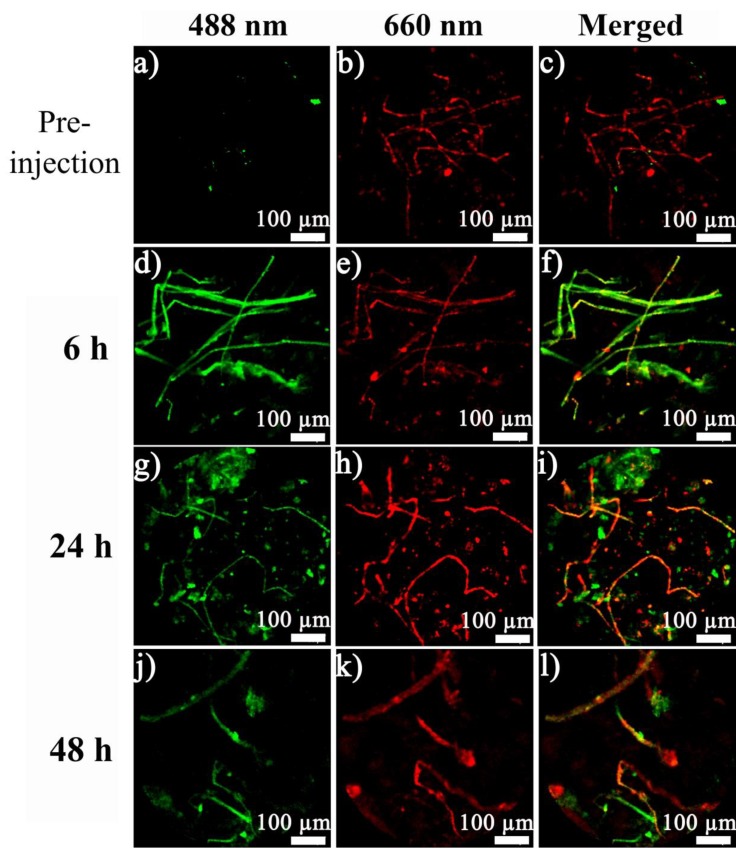
Fibered confocal fluorescence microscopic (FCFM) images of GQD-NPs (imaged under 488 nm excitation, green) and blood vessels (imaged by tail vein injection of Evans blue under 660 nm excitation, red) in tumor tissues over time.

Evans blue dye strongly binds to hemoglobin in blood, and is therefore widely used to track the presence of blood vessels [29]. As seen in Figure 4, prior to injection, a clear vascular system was displayed at the tumor site under excitation with the 660 nm laser. A few green fluorescence lights were seen under 488 nm, since some substances in the visible light band would also emit green fluorescence under visible-light excitation. GQDs were employed in the tracking of nanoparticles *in vivo*. When the GQD-NPs were injected for six hours, green fluorescence indicated the presence of GQD-NPs, while the blood vessels at the tumor site were still clearly visible (red). In the merged image, it could be seen that a large number of GQD-NPs apeared in the tumor blood vessels, and a small amount of GQD-NPs penetrated into the surrounding tissues through the tumor blood vessel wall. When the GQD-NPs were injected for 24 h, the blood vessels at the tumor site were still clearly visible (red), and there were still many GQD-NPs within the tumor blood vessels (green). As time passed (from 6 h to 24 h), a large amount of GQD-NPs passed through the tumor vessel wall and penetrated into the surrounding tissues as for the enhanced permeability and retention (EPR) effect, achieving enrichment in the tumor tissues. When the GQD-NPs were injected for 48 h, there were still fluorescence signals of the GQD-NPs (green) in the tumor tissues and the blood vessels, but the fluorescence signals in the tumor tissues were significantly weakened (relative to 24 h). This indicated that more GQD-NPs had been metabolized. Thus, reduced enrichment of the GQD-NPs at the tumor site was found. The results illustrated that by loading GQDs into nanoparticles, the dynamic changes of nanoparticles could be tracked *in vivo*.

## 4. Conclusions

The one-pot method of loading graphene quantum dots (GQDs) was proposed as a means to modify nanoparticles for fluorescent imaging. GQDs were loaded into the optical-magneto Fe_3_O_4_@PPy nanoparticles instead of on the surface. The morphology, components, optical and magnetic properties, biocompatibility and biomedical application of the GQD-loaded optical-magneto nanoparticles were investigated. The GQD-loaded optical-magneto nanoparticles performed as an excellent tacking agent, highlighting the potential application of GQDs in tracking the real-time distribution and metabolism of nanoparticles *in vivo*.

## Supplementary Materials

The following are available online at https://www.mdpi.com/1996-1944/12/13/2191/s1, Figure S1: Hydrodynamic size of GQDs-NPs nanocomposites.
