# Supplementary material for: Loading Graphene Quantum Dots into Optical-Magneto Nanoparticles for Real-Time Tracking In Vivo
Source: Materials (Basel). 2019 Jul 8;12(13):2191. doi: 10.3390/ma12132191 (PMC6650881; doi:10.3390/ma12132191)
Supplement: Supplementary file 1 [file materials-12-02191-s001.pdf]

materials

Communication

Loading graphene quantum dots into optical-magneto nanoparticles for real-time tracking in vivo

Yu Wang [1,2], Nan Xu [1,2], Yongkai He [1,2,3], Jingyun Wang [4], Dan Wang [1,2], Qin Gao [1,2], Siyu Xie [1,2], Yage Li [1,2], Ranran Zhang [3,*] and Qiang Cai [1,2,3,*]

[1] State key laboratory of New Ceramics and Fine Processing, School of Materials Science and Engineering, Tsinghua University, Beijing 100084, China
[2] Key Laboratory of Advanced Materials of Ministry of Education of China, Tsinghua University, Beijing100084, China
[3] Tsinghua Shenzhen International Graduate School, Tsinghua University, Shenzhen 518055, China
[4] Shenzhen Geim Graphene Center, Tsinghua-Berkeley Shenzhen Institute, Tsinghua University, Shenzhen 518055, China
* Correspondence: biomnano@163.com (R.Z.); caiqiang@mail.tsinghua.edu.cn (Q.C.)

Academic Editor: name
Received: date; Accepted: date; Published: date

Dynamic light scattering (DLS; Malvern Instruments Zetasizer Nano ZS90 ,UK) was employed to measure the hydrodynamic diameter of the nanocomposites and the result was shown in **Figure** S1 (supporting information). It could be seen that the hydrodynamic diameters of the nanocomposites distributed from 50 nm to 200 nm, which was consistent with the result of HRTEM.

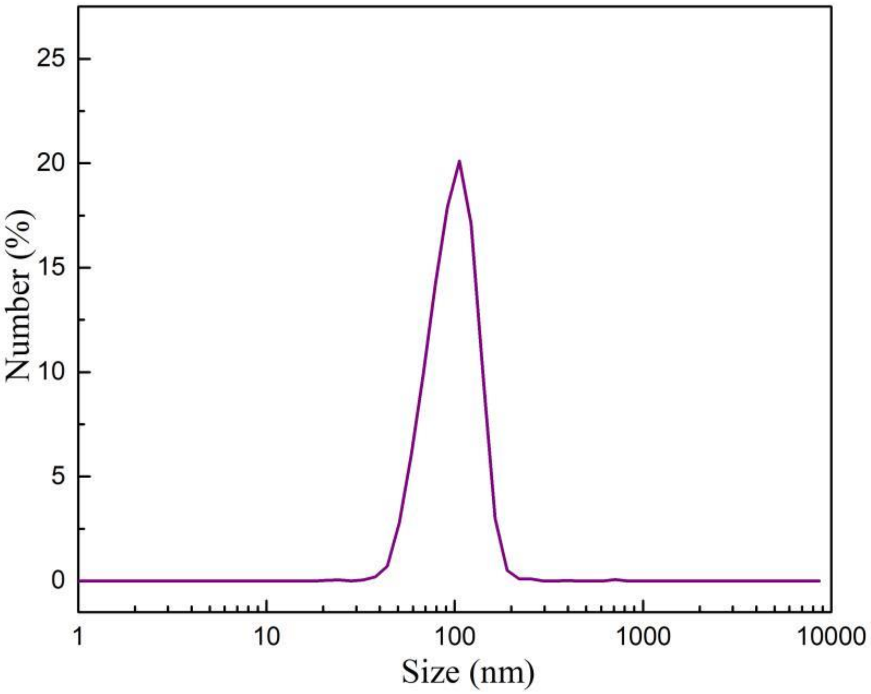

Figure S1. Hydrodynamic size of GQDs-NPs nanocomposites.
